# Childhood Eating and Feeding Disturbances

**DOI:** 10.3390/nu12040972

**Published:** 2020-04-01

**Authors:** Anja Hilbert

**Affiliations:** Integrated Research and Treatment Center AdiposityDiseases, Behavioral Medicine Research Unit, Department of Psychosomatic Medicine and Psychotherapy, University of Leipzig Medical Center, 04103 Leipzig, Germany; anja.hilbert@medizin.uni-leipzig.de; Tel.: +0049-341-9715360

**Keywords:** eating disorder, feeding disorder, anorexia nervosa, binge-eating disorder, avoidant/restrictive food intake disorder, loss of control eating, obesity

## Abstract

Eating and feeding disturbances are prevalent yet understudied health conditions in youth. They are characterized by aberrant eating behaviors, cognitive and emotional dysfunctions, and dysregulated body weight. The Diagnostic and Statistical Manual of Mental Disorders Fifth Edition defines several feeding and eating disorders with a common onset in youth; however, data on their clinical validity at young ages are lacking. Further non-normative eating behaviors exist, but their clinical relevance needs elucidation. This Special Issue compiles state-of-the-art reviews and empirical research on the presentation, development, course, and maintenance of diverse eating and feeding disturbances as a prerequisite for delineating evidence-based interventions for treatment and prevention.

## 1. Introduction

Eating and feeding disturbances in youth are understudied health conditions. They are characterized—to varying degrees—by non-normative eating behaviors, associated cognitive and emotional dysfunctions, and dysregulated body weight [1]. Despite a high prevalence of eating and feeding disturbances, affecting about 19.8% of youth ages 11–17 years [2], there is a lack of research on their presentation, development, course, and maintenance [3], which are prerequisites for delineating evidence-based interventions for treatment and prevention.

Anorexia nervosa, bulimia nervosa, and binge-eating disorder represent the specific eating disorders in the Diagnostic and Statistical Manual of Mental Disorders Fifth Edition (DSM-5; [1]). These disorders often show an early onset, mostly in adolescence [4], and result in significant impairments in health, psychosocial functioning, and quality of life. The hallmark feature of anorexia nervosa is a significantly low body weight, provoked by a restriction of energy intake relative to requirements. In contrast, the central characteristic of bulimia nervosa and binge-eating disorder are recurrent binge-eating episodes, defined as losing control over eating amounts of food that are objectively large. While attempts to prevent weight gain through inappropriate compensatory behaviors such as self-induced vomiting are characteristic of bulimia nervosa, binge-eating disorder does not present with a regular use of such behaviors.

Further feeding or eating disorders have newly been defined in or moved to the DSM-5 Feeding and Eating Disorders Section [1], including avoidant/restrictive food intake disorder, in addition to pica (persistent eating of nonnutritive substances) and rumination disorder (repeated regurgitation of food). Avoidant/restrictive food intake disorder is characterized by a persistent failure to meet appropriate nutritional or energy needs, based on a range of different motivations, such as a lack of interest in eating, but—as opposed to anorexia nervosa—not based on weight and shape concern. In contrast, other forms of non-normative eating behaviors with potential clinical significance exist that have not been included in the DSM-5, which is likely because their clinical validity is still unclear; for example, loss of control eating, involving a sense of losing control over eating subjectively or objectively large amounts of food and its proposed clinical variant of loss of control eating disorder [5].

The objective of this Special Issue on “Childhood Eating and Feeding Disturbances” was to aggregate clinically relevant research on these disturbances in youth. Two scientific reviews and eight empirical research articles were compiled on the clinical presentation, development, course, and maintenance of diverse eating and feeding disturbances.

## 2. Clinical Presentation, Course, and Maintenance of Anorexia Nervosa

Anorexia nervosa is increasingly prevalent in children from many countries; however, this disorder with an early onset has received scant attention in research. In her state-of-the-art review, Herpertz-Dahlmann [6] highlighted clinically relevant aspects for the age-adapted diagnosis and treatment of anorexia nervosa in childhood. As this disorder in childhood leads to substantial impairment in physical and mental health with a potential deleterious effect on later life, further research is warranted to better describe, diagnose, and treat this disorder, especially at young ages.

Jaite et al. [7] examined the sociodemographic and clinical profile of children versus adolescents with anorexia nervosa, presenting for inpatient treatment and enrolled in a large consecutive multicenter registry in Germany. Compared to adolescents, children with anorexia nervosa had a shorter duration of illness and higher body mass index percentiles at both admission and discharge, suggesting a more favorable outcome at a younger age, although long-term effects remain to be examined.

Olivo et al. [8] conducted a systematic review of functional magnetic resonance imaging studies in adolescents with anorexia nervosa; currently, there are no such studies at younger ages. Most evidence was found for an impairment in brain development through underweight and pubertal delay, involving an impaired cognitive flexibility, which may contribute to the maintenance of eating disorder symptoms. These and other findings were integrated into a neuroscientific pathogenic model, allowing the derivation of neuroscientifically informed interventions.

## 3. Clinical Presentation of Avoidant/Restrictive Food Intake Disorder

Clinical descriptions of avoidant/restrictive food intake disorder highlight a reduced dietary intake of a limited volume and variety of foods. For a systematic characterization of intake, Harshman et al. [9] evaluated the dietary variety in youth aged 9–22 years with this disorder using food records. Individuals with full or subthreshold avoidant/restrictive food intake disorder reported a lower consumption of vegetables or fruit, lower intake of protein, and relatedly, of specific vitamins (e.g., vitamins K and B12), but a higher intake of added sugars and total carbohydrates when compared to healthy controls. As this low dietary quality is likely to increase the risk for malnutrition, therapeutic approaches need to address an improvement in food variety to prevent nutritional deficiencies and associated sequelae.

Duncombe-Lowe et al. [10] investigated the clinical presentation of youth aged 8–18 years with avoidant/restrictive food intake disorder presenting at an eating disorder service. An acute symptom onset, which was more present in girls than in boys, was related to greater mental comorbidity (i.e., depression) and stronger tendencies of self-harm and suicidal ideation. In contrast, chronic symptom onset co-occurred with a higher body weight at admission. These results suggest differential care requirements for acute versus chronic patterns of symptom onset in avoidant/restrictive food intake disorder, including rapid care in patients with acute symptom onset for safety reasons. As in previous studies, high rates of overlapping symptom presentations of lack of interest in food, sensory sensitivities, and fear of aversive consequences were found in about half of patients, supporting a dimensional conceptualization of this disorder [11].

## 4. Clinical Presentation, Development, Course, and Maintenance of Non-Normative Eating Behaviors

Current research focuses on the neurocognitive profile underlying eating and feeding disturbances. For adolescent binge-eating disorder, previous evidence documented a biased processing of visual food stimuli [12] and impaired inhibitory control [13], likely contributing to binge-eating episode occurrence. In 9–16-year-old youth with loss of control eating, Biehl et al. [14] investigated the processing of distracting visual food stimuli during a Go/NoGo task via electroencephalography and found that in NoGo trials, mean P3 amplitudes were significantly higher when the distractor was a high-calorie food stimulus versus a neutral stimulus, while in Go trials, the loss of control group’s mean P3 amplitudes did not vary across distractor categories. The control group showed an inverse pattern. These results suggest an altered behavioral inhibition in response to food cues to already be present in subclinical child and adolescent loss of control eating.

While short sleep duration is a well-supported risk factor for childhood obesity [15], the precise associations with regard to non-normative eating behaviors are less clear. Using actigraphy, LeMay-Russell et al. [16] separately examined weekday and weekend sleep in 8–17-year-old youth. Weekday and weekend sleep showed differential associations with non-normative eating behaviors, in that shorter weekday sleep and greater weekend “catch-up” sleep were both associated with higher eating in the absence of hunger. Both associations were assumed to confer an increased risk of excess weight gain in the long term. Longitudinal research may ascertain the relevance of both sleep variability and eating in the absence of hunger as targets in obesity prevention.

Wentz et al. [17] examined the prevalence of eating disturbances in children with obesity and the differential effect of mental comorbidity, including attention deficit/hyperactivity disorder and autism spectrum disorder, and both were found to contribute to an increased risk of excess weight gain. In youth aged 5–16 years presenting to an obesity treatment service, eating disturbances including loss of control eating were more prevalent than in youth from the general population, which is in line with the literature. Interestingly, comorbidity with attention deficit/hyperactivity disorder and autism spectrum disorder did not impact these associations. These results are not suggestive of an overlap between eating disturbances and the investigated neurodevelopmental disorders in children and adolescents with obesity. In other words, unlike the evidence on body dissatisfaction as a shared risk factor [18], the results do not support this neurodevelopmental comorbidity as a shared risk factor between eating disturbances and obesity.

Parental feeding practices have been shown to be relevant for the development and maintenance of eating disturbances and overweight in youth across a wide age range; however, differential associations with specific symptoms of eating and weight disorders and the direction of the causal pathways are less clear. In a large population-based sample of children aged 8–13 years, Schmidt et al. [19] found that cross-sectionally, greater restrictive feeding by parents accounted for higher loss of control eating in older children and greater eating disorder psychopathology in younger children, while monitoring the child’s weight was associated with greater eating disorder psychopathology in older children only. The associations of restrictive feeding practices were particularly pronounced in children with higher weight status. Whether controlling parental feeding practices contribute to greater vulnerability regarding eating disturbances in children at higher weight needs confirmation in prospective research.

Parental feeding practices, for example, restrictive feeding, have been assumed to undermine children’s weight self-regulation. In a large longitudinal cohort of 2–12-year-old children, Eichler et al. [20] evaluated bidirectional associations between parental feeding practices and standardized body mass index (zBMI) at one-year intervals. Child zBMI predicted restrictive feeding practices, pressure to eat, and monitoring of the child’s food intake at multiple ages, while using food as a reward predicted child zBMI only from 4 to 5 years of age. These data suggest that parents adapt their feeding practices to child zBMI rather than influence it, which is in line with some previous research. The exception was on parental food-rewarding, which may have a detrimental effect on preschool children’s weight regulation, which is likely because it fosters non-normative eating behaviors, for example, greater responsiveness to food cues, craving, and emotional overeating.

## 5. Conclusions

Overall, the research collated in this Special Issue advances the understanding of eating and feeding disturbances in youth. It contributes to the validation of these prevalent yet understudied diagnoses as well as non-normative eating behaviors as their potential precursors, with consideration of associations with other health conditions such as obesity. Illness conceptualizations and specific clinical recommendations for the diagnosis and treatment are given, particularly for anorexia nervosa at different ages. Taken together, the presented research highlights the necessity of a developmentally sensitive approach to the investigation and prevention or early intervention of eating and feeding disturbances at young ages.
